# *ERBB2* (HER2) amplifications and co-occurring *KRAS* alterations in the circulating cell-free DNA of pancreatic ductal adenocarcinoma patients and response to HER2 inhibition

**DOI:** 10.3389/fonc.2024.1339302

**Published:** 2024-02-08

**Authors:** Afsaneh Barzi, Caroline M. Weipert, Carin R. Espenschied, Victoria M. Raymond, Andrea Wang-Gillam, Mohammad Amin Nezami, Eva J. Gordon, Daruka Mahadevan, Kabir Mody

**Affiliations:** ^1^Department of Medical Oncology & Therapeutics Research, City of Hope National Medical Center, Duarte, CA, United States; ^2^Guardant Health, Redwood City, CA, United States; ^3^Division of Oncology, Siteman Cancer Center, St. Louis, MO, United States; ^4^Orange Coast Medical Center of Hope, Newport Beach, CA, United States; ^5^Private Health Management, Inc., Los Angeles, CA, United States; ^6^Division of Hematology and Oncology, Department of Medicine, University of Texas Health, San Antonio, San Antonio, TX, United States; ^7^Division of Hematology-Oncology, Department of Medicine, Mayo Clinic, Jacksonville, FL, United States

**Keywords:** pancreatic cancer, HER2 amplification, ERBB2, ctDNA, liquid biopsy

## Abstract

**Purpose:**

Despite accumulating data regarding the genomic landscape of pancreatic ductal adenocarcinoma (PDAC), olaparib is the only biomarker-driven FDA-approved targeted therapy with a PDAC-specific approval. Treating *ERBB2*(HER2)-amplified PDAC with anti-HER2 therapy has been reported with mixed results. Most pancreatic adenocarcinomas have *KRAS* alterations, which have been shown to be a marker of resistance to HER2-targeted therapies in other malignancies, though the impact of these alterations in pancreatic cancer is unknown. We describe two cases of *ERBB2*-amplified pancreatic cancer patients treated with anti-HER2 therapy and provide data on the frequency of *ERBB2* amplifications and *KRAS* alterations identified by clinical circulating cell-free DNA testing.

**Methods:**

De-identified molecular test results for all patients with pancreatic cancer who received clinical cell-free circulating DNA analysis (Guardant360) between 06/2014 and 01/2018 were analyzed. Cell-free circulating DNA analysis included next-generation sequencing of up to 73 genes, including select small insertion/deletions, copy number amplifications, and fusions.

**Results:**

Of 1,791 patients with pancreatic adenocarcinoma, 36 (2.0%) had an *ERBB2* amplification, 26 (72.2%) of whom had a *KRAS* alteration. Treatment data were available for seven patients. Two were treated with anti-HER2 therapy after their cell-free circulating DNA result, with both benefiting from therapy, including one with a durable response to trastuzumab and no *KRAS* alteration detected until progression.

**Conclusion:**

Our case series illustrates that certain patients with *ERBB2*-amplified pancreatic adenocarcinoma may respond to anti-HER2 therapy and gain several months of prolonged survival. Our data suggests *KRAS* mutations as a possible mechanism of primary and acquired resistance to anti-HER2 therapy in pancreatic cancer. Additional studies are needed to clarify the role of *KRAS* in resistance to anti-HER2 therapy.

## 1 Introduction

Pancreatic ductal adenocarcinoma (PDAC) is notoriously challenging to diagnose and treat, with over 80% of patients having regional or distant metastases at diagnosis, and over 87% surviving less than five years (1). There is currently only one United States Food and Drug Administration (FDA)-approved biomarker-driven targeted therapy approved specifically for PDAC (the poly (ADP-ribose) polymerase (PARP) inhibitor olaparib), despite several PDAC sequencing studies identifying potential genomic targets, including homologous recombination deficiency (HRD) genes, *BRAF*, and receptor tyrosine kinases, such as *ERBB2*(HER2) (2–8).

Recent studies have identified *ERBB2*(HER2) amplifications in PDAC at rates of 1-7% (2–9). Targeted therapies are FDA-approved for *ERBB2*-amplified breast, colon, and gastric cancer (10–14). In the past, trastuzumab efficacy was examined in patients with *ERBB2* positive PDAC, as assessed by IHC, with confirmed response rates of 6% to 23.5%, but numbers were small and tumors were not assessed for co-occurring mutations and potential mechanisms of resistance (15, 16). In the more recent Know Your Tumor study, four *ERBB2*-amplified patients with PDAC received trastuzumab in combination with various drugs and observed responses ranging from one month to over 12 months (5). The phase II MyPathway trial included nine *ERBB2*-amplified patients with PDAC who were treated with trastuzumab and pertuzumab, of whom two achieved a partial response (11). Additional case reports have shown mixed results, with a *KRAS* wild-type, *ERBB2*-amplified patient with PDAC progressing within one month of treatment with trastuzumab emtansine in the fifth-line setting, and another patient achieving stable disease with trastuzumab and pertuzumab, followed by complete response when treated with immunotherapy, radiation, and trastuzumab deruxtecan in the third-line setting (4, 17). *KRAS* mutations have been seen at the time of primary and acquired resistance to anti-HER2 therapy in other cancer types, including colorectal and gastroesophageal cancer; however, the impact of *KRAS* mutations co-occurring with potentially targetable alterations in PDAC has not been defined (11, 18–20).

While mutations in potentially targetable genes in PDAC are individually rare, recent studies suggest that 10-20% of PDAC tumors may harbor therapeutically actionable alterations (4, 21). Comprehensive genomic profiling with next-generation sequencing (NGS) provides the opportunity to identify patients who may benefit from a targeted therapy approach, and analysis of circulating cell-free DNA (cfDNA) could be particularly useful in PDAC where tissue specimens are limited and patients often have less time to wait for genomic test results given the urgency to initiate treatment (22, 23). Herein, we describe two cases of patients with *ERBB2*-amplified PDAC treated with anti-HER2 therapy following their cfDNA result, and assess the frequency of *ERBB2* amplification and co-occurring *KRAS* mutations and/or amplification in clinical cfDNA NGS results in over 1,700 samples from patients with PDAC.

## 2 Materials and methods

### 2.1 Patients

We analyzed the Guardant360 deidentified database, containing results of patients who underwent clinical cfDNA testing between June 2014 and January 2018, and identified patients with a diagnosis of PDAC as reported by the ordering provider on the test requisition form. Patients with PDAC who had an *ERBB2* amplification detected on at least one cfDNA test were included for further analysis. This research was approved by the Advarra Institutional Review Board (IRB) for the generation of deidentified data sets for research purposes. For select patients, additional details regarding treatments and outcomes were obtained from the treating physician as per local IRB guidelines.

### 2.2 cfDNA analysis

Blood draw, shipment, plasma isolation and cfDNA extraction procedures for the clinical cfDNA assay used in this study have been previously described (24). Guardant360 is a CLIA-certified, College of American Pathologists-accredited, New York State Department of Health-approved cfDNA NGS assay with analytic and clinical validation reported (24). Point mutations were analyzed in 68 to 73 genes, small insertions and/or deletions (indels) in up to 23 genes, copy number amplifications (CNA) in up to 18 genes, and fusions in up to six genes, depending on the panel version performed (**Table 1**) (24).

**Table 1:** Genes and mutation types analyzed in cfDNA analysis (Guardant360 73 gene panel).

Point Mutations (SNVs)						
*AKT1*	*ALK*	***APC***	***AR***	*ARAF*	*ARID1A*	*ATM*
***BRAF***	***BRCA1***	***BRCA2***	*CCND1*	*CCND2*	*CCNE1*	*CDH1*
*CDK4*	*CDK6*	*CDKN2A*	*CTNNB1*	DDR2*	***EGFR***	***ERBB2***
*ESR1*	*EZH2*	*FBXW7*	*FGFR1*	*FGFR2*	*FGFR3*	*GATA3*
*GNA11*	*GNAQ*	*GNAS*	*HNF1A*	***HRAS***	*IDH1*	*IDH2*
*JAK2*	*JAK3*	***KIT***	***KRAS***	*MAP2K1*	*MAP2K2*	MAPK1*
MAPK3*	***MET***	*MLH1*	*MPL*	MTOR*	***MYC***	*NF1*
*NFE2L2*	*NOTCH1*	*NPM1*	***NRAS***	*NTRK1*	NTRK3*	*PDGFRA*
***PIK3CA***	*PTEN*	*PTPN11*	*RAF1*	RB1**	*RET*	*RHEB*
*RHOA*	*RIT1*	*ROS1*	*SMAD4*	*SMO*	***STK11***	*TERT*
***TP53***	TSC1**	*VHL*				
Indels						
*ATM*	*APC*	*ARID1A*	*BRCA1*	*BRCA2*	*CDH1*	*CDKN2A*
*EGFR*	ERBB2**	*GATA3*	*KIT*	*MET* Ex 14 skipping**	*MLH1*	*MTOR*
*NF1*	*PDGFRA*	*PTEN*	*RB1*	*SMAD4*	*STK11*	*TP53*
*TSC1*	*VHL*					
Amplifications						
*AR*	*BRAF*	CCND1**	CCND2**	*CCNE1*	*CDK4*	CDK6
*EGFR*	*ERBB2*	*FGFR1*	*FGFR2*	*KIT*	*KRAS*	*MET*
*MYC*	*PDGFRA*	*PIK3CA*	*RAF1*			
Fusions						
*ALK*	FGFR2**	FGFR3**	*RET*	*ROS1*	*NTRK1*	

**Bold** indicates complete exon coverage, otherwise critical exon coverage; Ex, exon.

*Genes/alterations not covered in 70-gene panel.

Genes included in 70-gene panel and removed for 73-gene panel: CDKN2B, SRC.

**Additional genes/alterations not covered in 68-gene panel.

### 2.3 Data analysis

cfDNA *ERBB2* amplifications were classified by copy number and category (1+/2+/3+) and assessed in comparison with co-occurring cfDNA identified *KRAS* alterations. At the time of this study, amplifications were reported on a semi-quantitative scale given that the absolute number of copies in circulation is dependent on both tumor fraction and the magnitude of amplifications. The 1+ category applied to amplification magnitude in the lower 50^th^ percentile of samples with amplifications, 2+ applied to amplification magnitude in the 50^th^ to 90^th^ percentile, and 3+ applied to amplification magnitude in the top 10^th^ percentile. *KRAS* alterations were categorized as amplifications or single nucleotide variants (SNVs). All characterized pathogenic *KRAS* SNVs were included. For patients with cfDNA analysis at multiple time points, *ERBB2* amplifications and *KRAS* alterations were assessed for changes over time.

## 3 Results

**Figure 1:**
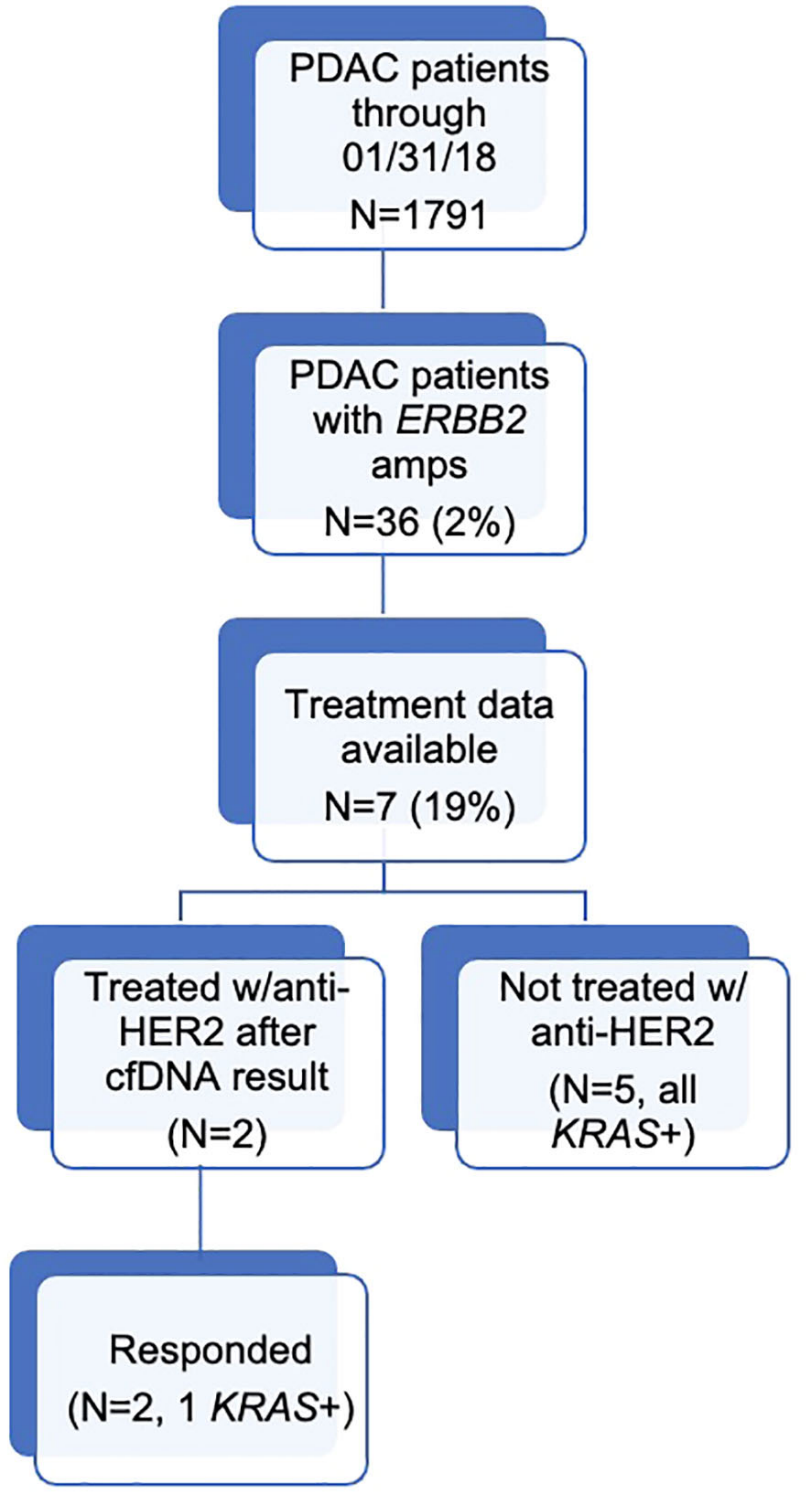
Diagram outlining the number of patients in each study category.

During the study period, 1,791 patients with PDAC had ≥1 alteration detected via cfDNA analysis, 36 (2.0%) of whom had an *ERBB2* amplification (**Figure 1**). The cohort of patients with *ERBB2*-amplified PDAC was 44.4% female with a mean (median) age of 66.3 (65.5) years (range 32-91; **Table 2**). Among the 21 patients for whom date of initial diagnosis was available, the mean (median) time from diagnosis to blood draw for liquid biopsy was 582.4 (366.5) days (range 7-2,452). Most patients were receiving systemic therapy at the time of blood draw for liquid biopsy (**Table 2**).

**Table 2:** Patient Demographics.

	N (%/range)
Patients	36
Gender	
Female Male	16 (44.4) 20 (55.6)
Age (years)	
Mean Median	66.3 (32–91) 65.5 (32–91)
Number of cfDNA time points	
1 2 3 4	31 (86.1) 3 (8.3) 1 (2.8) 1 (2.8)
cfDNA panel version*	
68-gene panel 70-gene panel 73-gene panel	3 (8.3) 16 (44.5) 17 (47.2)
Time from diagnosis to cfDNA blood draw (days, N=21)	
Mean Median	582.4 (7–2452) 366.5 (7-2452)
Current treatment regimen**	
Gemcitabine + Nab-Paclitaxel FOLFIRINOX FOLFOX FOLFIRINOX + Capecitabine FOLFIRINOX + Gemcitabine Irinotecan Oxaliplatin + Capecitabine Targeted therapy on trial None—newly diagnosed None—other/unknown reason Unknown	5 (13.8) 4 (11.1) 1 (2.8) 1 (2.8) 1 (2.8) 1 (2.8) 1 (2.8) 1 (2.8) 2 (5.6) 3 (8.3) 16 (44.4)

*For patients with multiple time points spanning different panel versions, the largest panel was counted.

**Current treatment regimen at the time of blood draw for liquid biopsy. If more than one time point, treatment at the time of the 1^st^ blood draw was recorded.

Among patients with *ERBB2* amplifications (N=36), 19 (52.8%) were 1+, 15 (41.7%) were 2+, and 2 (5.6%) were 3+ (**Table 2**). Of these, 26 (72.2%) also had a *KRAS* mutation and/or amplification, with 14 of 26 patients having co-occurring *KRAS* SNV(s) without *KRAS* amplification (**Table 3**).

**Table 3:** *ERBB2* amplification level (N=36) and co-occurring *KRAS* alterations.

	N (%)
*ERBB2* 3+ amplification *KRAS* SNV *KRAS* amplification *KRAS* SNV and amplification	2 (5.6) - - 2 (5.6)
*ERBB2* 2+ amplification *KRAS* SNV *KRAS* amplification *KRAS* SNV and amplification	15 (41.7) 6 (16.7) 2 (5.6) 3 (8.3)
*ERBB2* 1+ amplification *KRAS* SNV(s) *KRAS* amplification *KRAS* SNV and amplification	19 (52.8) 8 (22.2) 1 (2.8) 4 (11.1)
All cases with co-occurring *KRAS* alterations	26 (72.2)

For patients with more than one cfDNA time point, the time point where the ERBB2 amp and the most KRAS alterations were present was considered. Only pathogenic KRAS SNVs were considered. KRAS variants of uncertain significance were excluded.

SNV, single nucleotide variant.

**Figure 2:**
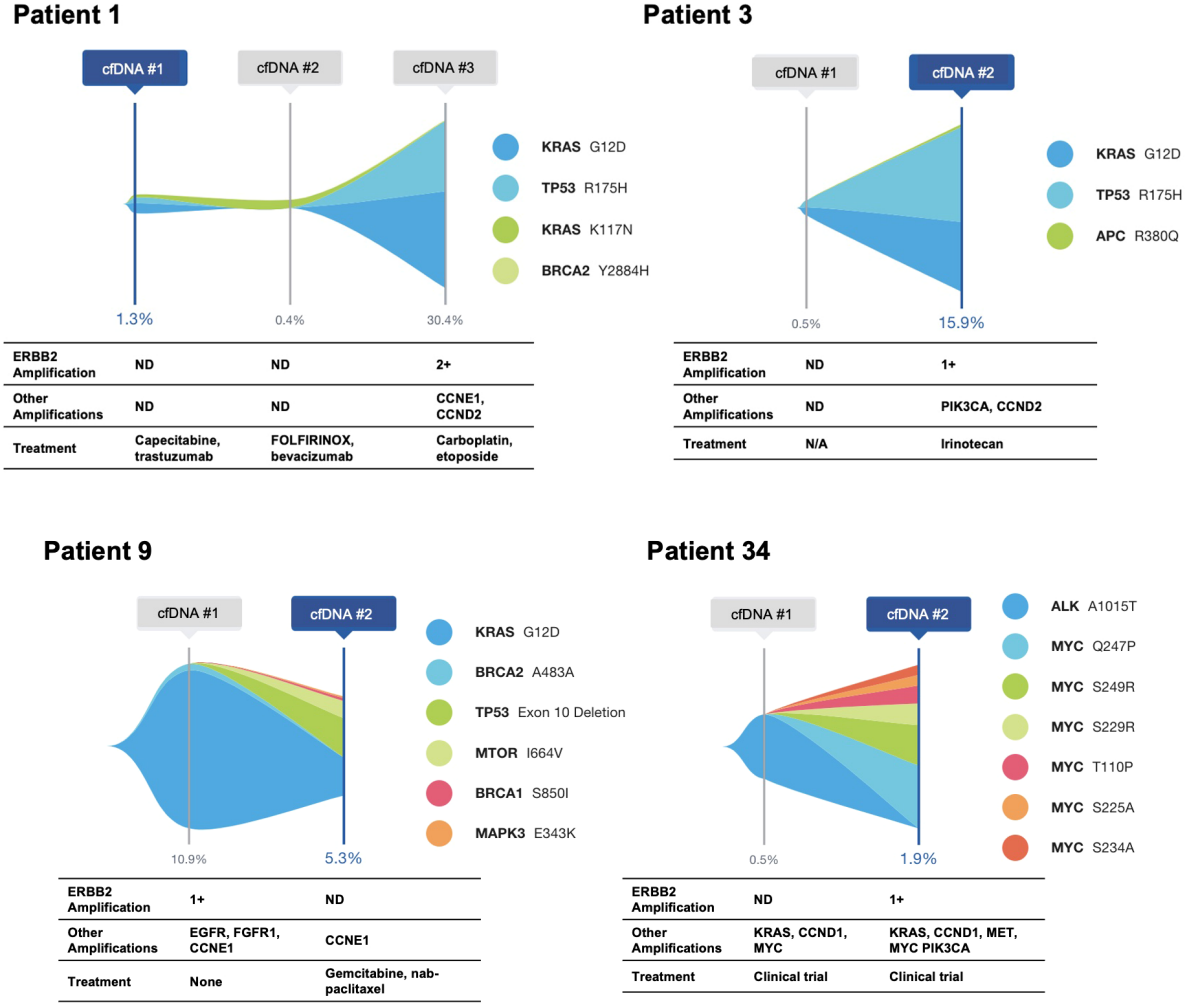
Serial cfDNA time points for patients with ERBB2 amplification identified in at least a single time point. Single nucleotide variants and insertion/deletion alterations are illustrated, with amplifications and treatment at the time of the cfDNA draw shown below.

**Figure 3:**
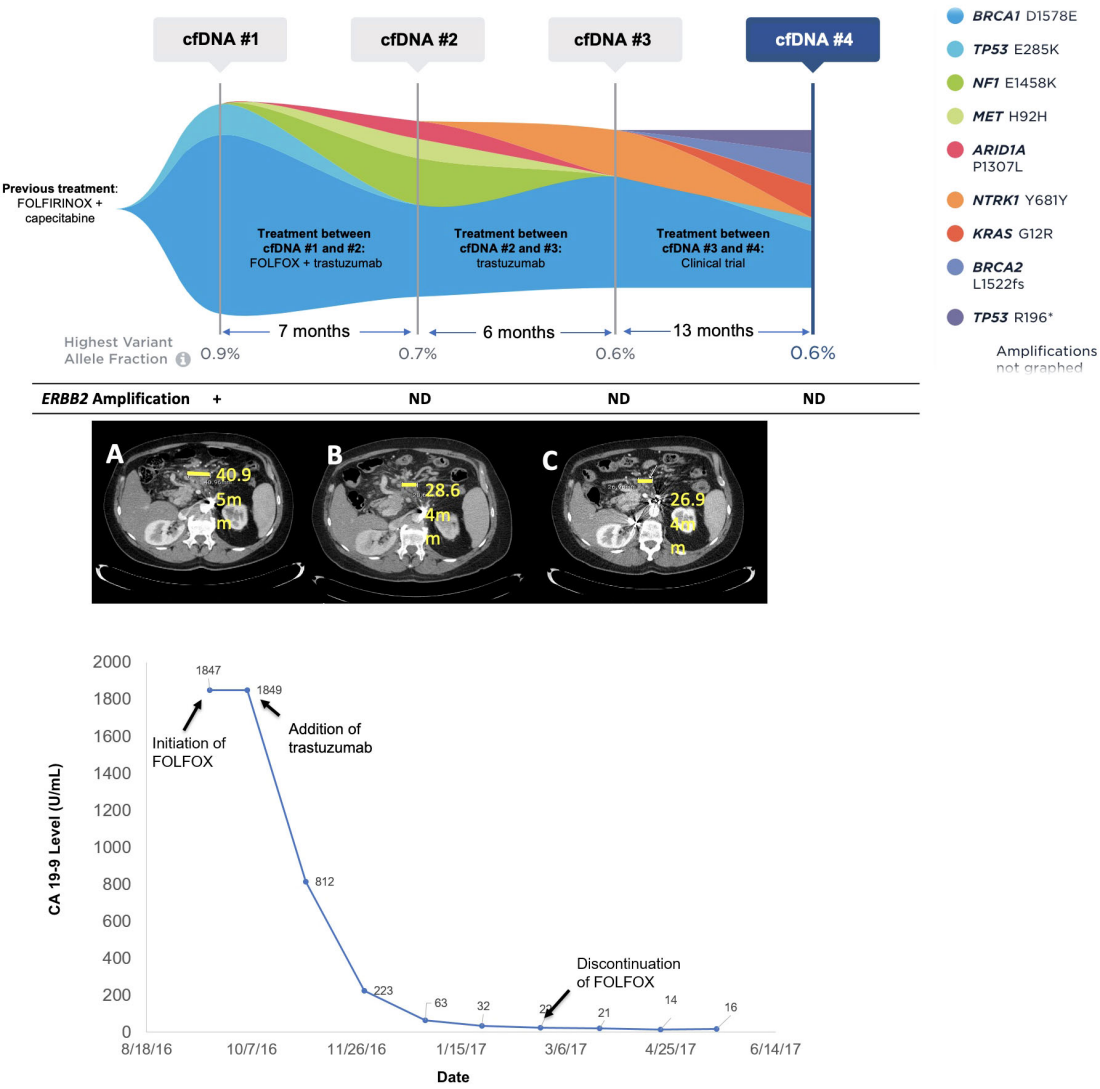
Molecular and clinical response to anti-HER2 therapy. Changes in mutations identified and variant allele fraction in Patient 17 as identified by cfDNA analysis along with corresponding systemic therapy and imaging results. The CA19-9 levels for Patient 17 over time corresponding to the systemic therapy and molecular results are also shown. Imaging shows CT scans at initation of FOLFOX **(A)**, discontinuation of FOLOX **(B)**, and three months after trastuzumab monotherapy **(C)**.

Five patients with *ERBB2*-amplified PDAC had cfDNA analysis at multiple time points (**Figure 2**; Patient 17 shown in **Figure 3**). For all five patients, the *ERBB2* amplification was detected at a single time point, and all had *KRAS* alterations at one or more time points. Three patients had at least one activating *KRAS* alteration and/or *KRAS* amplification detected in all samples, with the *ERBB2* amplification occurring in the last sample, notably corresponding to an increase in tumor shed, as measured via the maximum variant allele frequency (maxVAF) in the sample. One patient had a *KRAS* alteration and an *ERBB2* amplification detected in their first sample, with persistence of the *KRAS* alteration in their second sample but loss of the amplification. One patient (Patient 17 below) had *ERBB2* amplification detected in their first sample, with neither *ERBB2* amplification nor *KRAS* alterations detected in two subsequent blood draws, and a *KRAS* mutation detected in the fourth blood draw without identification of the *ERBB2* amplification.

Treatment data were available for seven patients, two of whom were treated with anti-HER2 therapy following their cfDNA result, with both benefiting from therapy. Patient 5 was a 56- year-old male with PDAC progressing after treatment with FOLFOX. cfDNA analysis identified an *ERBB2* amplification at 2.3 copies (1+) as well as *SMAD4* R361C at 35.1% VAF, *KRAS* G12D at 35.0% VAF, *TP53* Q144* at 31.7% VAF, *AR* T878S at 0.2% VAF, and *CDK6* amplification at 2.5 copies (1+). He was treated with the HER2/EGFR tyrosine kinase inhibitor afatinib in the second-line setting for one month and had stable disease with improved quality of life but was not a candidate for continued anti-HER2 or other subsequent therapy.

Patient 17 was a 64- year-old male, originally diagnosed with PDAC with metastases to the retroperitoneal lymph nodes. He was treated with first-line FOLFIRINOX and capecitabine maintenance for two years. At progression, liquid biopsy was ordered and was positive for an *ERBB2* amplification at 2.2 copies (1+), *TP53* E285K at 0.1% VAF, and a *BRCA1* variant of uncertain significance (VUS) at 0.9% VAF (**Figure 3**). The patient was initiated on FOLFOX, and trastuzumab was added soon afterward. The patient experienced clinical improvement and significant decline in CA19-9 (1,847 to 22 U/mL) over a four-month period (**Figure 3**). Only VUS and synonymous variants were detected on a repeat liquid biopsy, with a maxVAF of 0.7% (**Figure 3**). FOLFOX was discontinued after about four months and the patient remained on trastuzumab alone with further clinical improvement and decrease in CA19-9 (16 U/mL). After 6 months on trastuzumab, the patient began to experience progression, and a third cfDNA test continued to only show VUS and synonymous variants with a maxVAF of 0.6%. FOLFOX was re-introduced with no biochemical or radiographic response. The patient then went on a clinical trial with an immunotherapy combination and did well on this trial. A final cfDNA test taken approximately one year after the previous test demonstrated the emergence of *KRAS* G12R at 0.3% VAF, *BRCA2* L1522fs at 0.3% VAF, *TP53* R196* at 0.2% VAF, and *TP53* E285K at 0.1% VAF, with the *ERBB2* amplification still not detected (**Figure 3**).

## 4 Discussion

Therapeutic options in patients with metastatic PDAC are limited. Patients with *KRAS* wildtype disease may have a different biology that may be more amenable to targeted therapies, but *KRAS* wildtype disease is present in a minority of patients with PDAC. Advancement of targeted therapies in PDAC has been challenging, with only one FDA-approved biomarker-directed targeted therapy (olaparib) currently available with a PDAC-specific approval. *KRAS* is the most frequently mutated gene in PDAC (70-95%), and until recently, was not generally considered a therapeutically targetable alteration (6, 25–28). Historic trials evaluating *KRAS* positive PDAC have primarily focused on a variety of MEK inhibitors and have shown limited benefit (29). More recently, the development of drugs specifically targeting *KRAS* G12C alterations, known to be present in a minority of PDAC cases, have shown promise (30, 31). Other *KRAS* alterations, such as G12D are much more common in PDAC, and drugs targeting other non-G12C alterations are in development (32).

Outside of *KRAS*, the efficacy of other targeted therapies continues to be explored in PDAC. Germline and/or somatic mutations in HRD genes may be present in up to 20% of advanced PDAC tumors (2). While a study of veliparib in patients with PDAC and germline pathogenic *BRCA1/2* or *PALB2* mutations had no confirmed responses, a study of rucaparib in patients with germline or somatic *BRCA1/2* mutations had a 16% objective response rate (ORR) and 32% disease control rate (DCR) (33, 34). Additionally, a trial of olaparib in PDAC achieved a 22% ORR and 57% DCR, and the phase III POLO trial evaluating olaparib maintenance therapy versus placebo in patients with metastatic PDAC and germline *BRCA1/2* mutations found significantly longer progression free survival (PFS) for olaparib versus placebo, resulting in its FDA approval (35–38). Additional targetable biomarkers in PDAC include *NRG1* fusions, microsatellite instability (MSI), and *BRAF* V600E (39–47). The cumulative results of trials examining these biomarkers suggest identification of PDAC patients with targetable alterations, even if they are rare, may open up therapy options to patients who may have limited other options.

While studies have identified *ERBB2*(HER2) amplifications in PDAC at rates of 1-7%, most have shown mixed responses to HER2-targeted therapies in *ERBB2* positive PDAC patients, with variable documentation of co-occurring RAS mutations (2–9, 15, 16). In the Know Your Tumor study, four *ERBB2*-amplified patients with PDAC received trastuzumab in combination with various drugs and observed responses ranging from one month to over 12 months (5). The phase II MyPathway trial included nine *ERBB2*-amplified patients with PDAC who were treated with trastuzumab and pertuzumab, of whom two achieved a partial response (11). Additional trials examining anti-HER2 therapy in PDAC, like the TAPUR trial, have yet to read out (48, 49). Among this clinical laboratory database of 1,791 patients with PDAC, *ERBB2* amplifications were seen at rates consistent with previous, smaller, studies (2.0%), with the majority also harboring at least one *KRAS* activating alteration. Notably, we report a case in which a patient with *ERBB2*-amplified PDAC (in the absence of co-occurring *KRAS* mutations) responded to anti-HER2 therapy for several months with clinical improvement and decline of tumor markers, before demonstrating a *KRAS* alteration newly detected by cfDNA and loss of *ERBB2* amplification via cfDNA after progression. Of note, this patient had relatively low tumor shed throughout their disease course (maxVAF at each time point did not exceed 1%), and thus it is difficult to say exactly when the patient acquired the *KRAS* G12R alteration as it was only seen in cfDNA approximately one year following trastuzumab discontinuation. Acquired *KRAS* alterations have been seen at the time of progression on trastuzumab in other cancer types (18). In contrast, a patient with co-occurring *KRAS* G12D and *ERBB2* amplification prior to anti-HER2 therapy initiation achieved stable disease for one month.

The presence of *KRAS* alterations at the time of resistance to anti-HER2 therapy is consistent with recent data from a clinical trial of trastuzumab and lapatinib in metastatic colorectal cancer (19). In that study, RAS and RAF alterations were detected at baseline in the plasma of 6/7 (86%) patients refractory to anti-HER2 therapy, but in only 3/22 (14%) patients who benefited from anti-HER2 therapy. *KRAS* mutations were subsequently identified at progression in two patients who initially had SD and one with an initial PR (19). Notably, the MyPathway HER2 basket trial examining use of trastuzumab plus pertuzumab in *ERBB2*-amplified patients across solid tumors stratified patients by *KRAS* status, and showed that patients with a *KRAS* mutation had an ORR of 3.8% and DCR of 3.8%, compared to an ORR of 25.6% and DCR of 49.7% in patients who were *KRAS* wildtype, suggesting co-occurring *KRAS* mutations had a significant impact on likelihood of response (20). Taken together, these reports and our cases suggest that certain patients with *ERBB2*-amplified PDAC do benefit from anti-HER2 therapy. Though limited in size, our results are consistent with previous work suggesting that *KRAS* mutations may function as primary or acquired resistance to anti-HER2 therapy, similar to what has been observed in colorectal and gastroesophageal cancer.

Of note, two HER2-directed antibody-drug conjugates have now been FDA-approved in metastatic *ERBB2*-amplified breast cancer and are being explored in various other cancer types (50). These drugs have demonstrated significant improvements in patient outcomes in *ERBB2*-amplified breast cancer patients, and in the case of trastuzumab deruxtecan, are also approved for patients with “HER2-low” disease (50). Pan-cancer trials of these drugs have so far included a limited number of PDAC patients, with the phase II DESTINY-PanTumour02 trial including 25 PDAC patients, with three patients having response via independent central assessment, and the phase II KAMELEON trial including four PDAC patients, of whom one had a partial response (51, 52). Further exploration of the potential of these HER2-targeted antibody-drug conjugates in the PDAC population are needed.

Detection of copy number amplification via cfDNA is dependent on two factors: 1) the degree of tumor shed, and 2) the level of amplification. Thus, a low-level amplification may not be detected even in a patient with a high-degree of tumor shed (53). In each case where *ERBB2* amplification was not originally detected, the sample with the amplification detected had a higher maxVAF, indicative of increased tumor shed. Thus, we cannot rule-out the possibility that the *ERBB2* amplification was present in earlier samples but was occurring below the assay’s limit of detection. We also saw loss of *ERBB2* expression in patients who were originally *ERBB2*-amplified, and in one of these instances the patient (Patient 17) was known to have been treated with anti-HER2 therapy. Loss of HER2 expression following anti-HER2 therapy has been reported in other cancer types, and thus in some instances may explain loss of HER2 expression over time (54–56). Attempts to account for the degree of tumor shed when examining plasma-based copy number calls are ongoing, but it remains a challenge to clearly account for tumor evolution/heterogeneity, treatment effects, and tumor shed when assessing changes in the amplification status of a particular tumor, especially when dealing with low and medium-level amplifications (53).

Overall, review of patients with serial cfDNA samples demonstrate tumor evolution in response to therapy, illustrating a well-known cause of tumor heterogeneity. Previous studies using rapid autopsy sampling of multiple metastatic sites have demonstrated that the molecular makeup of the primary tumor versus each metastatic site can vary (57). Thus, in the setting of disease progression, tissue biopsies may be limited in their ability to fully capture acquired resistance mutations unless multiple metastatic sites are biopsied at multiple time points, which is often not feasible. The difficulty of repeating tissue biopsies in patients progressing on therapy is especially acute in PDAC, where rapid clinical deterioration can create additional challenges to successful tissue biopsy (22). Liquid biopsy is much less invasive and has a demonstrated ability to capture a global picture of the alterations present throughout a patient’s disease burden, and thus may be optimally suited for tracking disease response and development of acquired resistance alterations leading to disease progression (58).

There are several limitations to our study, including the fact that it is a retrospective analysis, and our knowledge of patient clinical history is limited in most cases to what is provided by the ordering provider on the test acquisition form, which may not be wholly accurate and does not include orthogonal molecular testing information. Given this, we can only comment on the response to anti-HER2 therapy for a limited number of patients. For most of the patients with serial samples, we can only make educated guesses about why we see fluctuations in the appearance of *ERBB2* amplification and/or *KRAS* activating mutations based on the degree of tumor shed, as detailed above. Additionally, it is possible that there are inherent biases in the cohort of patients selected to undergo testing via cfDNA by their treating physician (e.g., they could have more aggressive disease and/or have progressed through more lines of therapy). As such, the alterations seen here may not reflect the molecular landscape seen in a treatment naïve patient population and the cohort may not reflect the broader PDAC population.

In conclusion, this analysis of over 1,700 PDAC samples from patients undergoing clinical cfDNA testing demonstrates the utility of cfDNA in detecting targetable alterations in this patient population. Additionally, this case series of patients treated with anti-HER2 therapy based on *ERBB2*(HER2) amplification detected via cfDNA provides additional evidence to suggest that cfDNA may be an adequate tool to detect *ERBB2*(HER2) amplifications and identify patients who may benefit from anti-HER2 therapies, as demonstrated here by the patient who responded to anti-HER2 therapy for several months. Notably, there are a growing number of anti-HER2 therapies available, including multiple antibody-drug conjugates, meaning identification of these patients may become more relevant in the future (59). The importance of molecular testing in identifying patients with rare, but targetable, alterations across cancer types has been highlighted a number of times, and seems particularly relevant in PDAC given the lack of treatment options available and often rapid disease progression (22, 23). This case series supports the idea that co-occurring alterations may play a key role in determining which patients respond to targeted therapy or not, suggesting *KRAS* as a possible mechanism of innate and acquired resistance, and further demonstrating the need for patients to undergo comprehensive molecular profiling to identify optimal therapy. Given the well-known difficulties of obtaining tissue biopsy in PDAC, particularly following disease progression, cfDNA offers an attractive alternative (22). Moreover, cfDNA has a unique advantage in its ability to capture temporal and spatial tumor heterogeneity, both well-established causes of disease progression and mixed responses to treatment (58). This ability may be uniquely helpful in PDAC, as patients can progress quickly through lines of therapy and historic tissue biopsies may not accurately reflect the current molecular landscape, as shown here by the tumor evolution seen in serial cfDNA samples. Further studies reporting co-occurring mutations and clinical outcomes are needed to better clarify the role of *KRAS* and other potential mechanisms of resistance to anti-HER2 therapy to allow for the identification of PDAC patients most likely to benefit from this treatment, including further exploration of how molecular testing, applied more broadly in patients with PDAC, could aid in getting more patients with PDAC onto a growing number of targeted therapies.

## Data availability statement

The datasets presented in this article are not readily available because they are derived from commercial testing. This data can be made available under a fully executed data use agreement. Requests to access the datasets should be directed to cweipert@guardanthealth.com.

## Ethics statement

The studies involving humans were approved by the Advarra Institutional Review Board (IRB) for the generation of deidentified data sets for research purposes. For select patients, additional details regarding treatments and outcomes were obtained from the treating physician as per local IRB guidelines. The studies were conducted in accordance with the local legislation and institutional requirements. Written informed consent for participation was not required from the participants or the participants’ legal guardians/next of kin in accordance with the national legislation and institutional requirements.

## Author contributions

AB: Conceptualization, Methodology, Supervision, Writing – original draft, Writing – review & editing. CW: Formal analysis, Methodology, Writing – original draft, Writing – review & editing. CE: Conceptualization, Data curation, Formal analysis, Investigation, Methodology, Writing – original draft, Writing – review & editing. VR: Conceptualization, Methodology, Supervision, Writing – review & editing. AW-G: Data curation, Writing – review & editing. MN: Data curation, Writing – review & editing. EG: Data curation, Writing – review & editing. DM: Data curation, Writing – review & editing. KM: Data curation, Writing – review & editing.
